# Assessing the financial burden on patients and their households attending hospital clinics: a pilot cross-sectional study

**DOI:** 10.1186/s12913-025-13503-0

**Published:** 2025-10-22

**Authors:** Louise Rabbitt, James Curneen, Anna Hobbins, Patrick Gillespie, Michael Conall Dennedy

**Affiliations:** 1 Discipline of Pharmacology and Therapeutics, School of Medicine, University of Galway, Galway, Ireland; 2 Galway University Hospitals, Saolta University Healthcare Group, Galway, Ireland; 3 Research Ireland Centre for Medical Devices (CÚRAM 13/RC/2073_P2), Galway, Ireland; 4 Health Economics & Policy Analysis Centre (HEPAC), Institute for Lifecourse & Society (ILAS), University of Galway, Galway, Ireland; 5 Floor 3, Lambe Institute for Translational Research, School of Medicine, University of Galway, Newcastle Road, Galway, Ireland

**Keywords:** Cost analysis, Health economics, Cost of care, Treatment burden, Care burden, Out of pocket expenses, Outpatient consultations, Healthcare accessibility

## Abstract

**Background:**

The financial burden on patients in terms of private out-of-pocket personal costs related to accessing healthcare services has become a growing concern internationally. While healthcare systems and access arrangements differ internationally, private cost to patients and their households remains an under-researched area. The private out-of-pocket costs of attending hospital outpatient consultations in Ireland has not been previously established.

**Methods:**

We developed a data collection tool to measure the private costs to patients of attending hospital outpatient consultations. Resource items included travel time and expenses, missed work, need for accompanying carers, and care needs for dependents, in addition to demographic details. In the pilot study, the questionnaire was completed by 42 patients attending several hospital outpatient departments in a large teaching public hospital in the west of Ireland.

**Results:**

The pilot study demonstrated the questionnaire’s feasibility and acceptability. Estimates of private costs per visit showed considerable variability, with a median cost per patient of €131 (Inter Quartile Range €142; range €22-€370). Participants reported attending on average 4 appointments per year (mean 4.26, range 1–20, SD 3.7), giving a mean annual cost of attending hospital clinics of €559 per person. On average, participants spent 1.2 h (range 0.1-3.0) travelling to their appointment. Fourteen participants (33%) reported missing work, while 18 (43%) required an accompanying carer, of whom four carers were paid.

**Conclusions:**

Private costs related to attending outpatient clinics are not insignificant. Formal validation of the data collection tool is required, alongside further studies involving larger, more diverse participant samples to better quantify the financial implications for patients attending outpatient consultations.

**Registration:**

The study protocol was pre-registered using the Center for Open Science registration portal (08 March 2024).

**Supplementary Information:**

The online version contains supplementary material available at 10.1186/s12913-025-13503-0.

## Background

Equity of access to healthcare is a key objective of health policy in Ireland and internationally [1, 2]. Relatedly, projected increases in the demand for health and social care services are a major concern for healthcare providers, insurers, governments and patients worldwide [3, 4]; which will have direct implications for the equity of access to services in increasingly constrained health and social care budgets. While health and social care systems and eligibility arrangements for free services differ from country to country, private finance and patient out-of-pocket payments play a key role in enabling patients to access services. In this context, the financial burden that falls on patients in terms of private out-of-pocket personal expenses related to accessing care is an important equity concern.

The Irish healthcare system provides an interesting test case in this regard, given that it involves a complex mix of public and private health and social care finance and provision. Public finance accounts for 75% of total health expenditure, while out-of-pocket payments and voluntary health insurance accounts for 11.7% and 14% respectively [5]. Public hospital care is provided at no or reduced cost for all residents, though 46% of people purchase voluntary private health insurance [5]. Hospital outpatient care accounts for a quarter of health spending, and is provided free of charge to all residents [5]. Public hospitals receive 70% of their public funding via an activity-based funding (ABF) reimbursement model and the remainder on the basis of historic block and population-based funding [6]. The introduction of an ABF reimbursement mechanism in Ireland reflects a policy commitment to a more efficient hospital system [6]. 

The Irish public hospital system, while aiming to provide services free at the point of use still places a considerable financial strain on patients in the form of costs directly incurred by the patient and their household related to their hospital visit. Further, the public hospital system in Ireland has been facing significant resourcing challenges, resulting in long waiting times and overcrowding, which have the potential to further exacerbate equity concerns through the relative imbalance in the distribution of such costs across the patient population. That said, little is known about the private costs incurred by patients relating to accessing hospital care in Ireland. A recent cost analysis completed by our group used micro-costing methods to estimate the cost from the healthcare perspective of delivering care in the specialist hypertension outpatient clinic [7]. While informative, this and other similar studies overlook the private cost of a clinic attendance from the patient perspective [8]. Importantly, such private costs have been cited as a reason for non-attendance, which has been estimated to cost €20 million per annum in Ireland [9]. 

The principal objective of this study was to quantify the economic burden in terms of the private costs experienced by patients and their households from attending hospital outpatient clinics in two public hospitals within the Saolta University Healthcare Group in Ireland (University Hospital Galway and Merlin Park Hospital). These hospitals provide general and specialised services to a large catchment area of approximately 1 million citizens, spanning urban and rural populations. We developed a bespoke data collection tool and conducted a pilot study to estimate the costs incurred by patients attending these centres. We use the term ‘private costs’ to encapsulate the personal, out-of-pocket financial burden borne directly by patients, to distinguish these from public or institutional expenditure. By quantifying these costs and identifying their determinants, this study aims to provide valuable insights to policymakers, healthcare providers, and the public. These findings will inform future research on this important topic, provide a perspective that has international relevance, and ultimately work towards more equitable and accessible healthcare systems.

## Methods

The aim of this study was twofold: (i) to develop a data collection tool to investigate the private cost experienced by patients from attending outpatient clinics, and (ii) to evaluate its acceptability and feasibility through a pilot cross-sectional questionnaire study and provide preliminary estimates of private patient costs. Ethical approval was granted by the GUH Clinical Research Ethics Committee (CA3140 2nd February 2024).

### Data collection tool development

To develop a suitable data collection tool, the research team identified the data points required to achieve the study objectives. The objective of the tool was to identify and measure all relevant private resource activity related to attending hospital clinic attendance. Due to the limited availability of validated tools for measuring health-related costs incurred by patients [10], items were selected based on face validity, drawing from published literature and prior studies conducted by the group [11]. Additionally, items addressing treatment burden—defined as the impact of healthcare on patient functioning and well-being, excluding specific treatment side effects [12]—were included to capture patients’ perceived burden of attending outpatient appointments, distinct from their financial costs.

The study concept was developed with a Public and Patient Involvement (PPI) panel, who endorsed its relevance to their experiences of appointment-related burdens. PPI provides a robust, evidence-based model for involving patients in shaping healthcare research to ensure it is relevant and applicable to the public [13]. The PPI panel reviewed the questionnaire before deployment, confirming its acceptability and comprehensiveness. Their feedback led to minor refinements in wording and layout to improve clarity and accessibility.

For items relating to travel time, out-of-pocket expenses and similar costs, the scope was limited to patients’ expenses for that particular visit, to minimise recall bias [14]. Questions were ordered by relevance to the research question to prioritize key data in case of incomplete responses. The questionnaire is available in Appendix A.

### Pilot study and cost analysis

As this was an exploratory pilot study, no formal sample size calculation was performed. Based on published literature, a sample size of 40 was estimated to be adequate to assess feasibility and acceptability, while being achievable within the resources available to the study group. A maximum variation sampling strategy was adopted; an opportunistic sample of participants were consecutively recruited from the waiting rooms of a number of outpatient clinics in GUH and Merlin Park Hospital over a two-week period in February 2024. Clinics included diabetes, neurology, respiratory, gastroenterology, hepatology, infectious diseases, lipidology, hypertension and nephrology. Two researchers (LR and JC) approached participants in the waiting rooms to introduce themselves and obtained informed consent. Survey responses were collected electronically using an iPad.

The cost analysis comprised on valuing each resource activity identified and measured by their respective unit costs. A total private patient cost was generated by summing all the individual private resource cost components. Unit costs were identified from public sources and following guidance for the conduct of health economic evaluation in Ireland [15]. The value of one working hour was set at the national mean gross hourly wage (€28.43) [16]. The time of participants who reported missing work to attend was valued at this level. For all other participants, including retirees, their time was considered leisure time. The value of the time of accompanying carers (if not paid directly by the patient) was calculated similarly. The value of leisure time was set at 35% of the national hourly gross wage, based on existing evidence and applicable standards [17]. Travel costs were calculated using nationally agreed public service employee travel and subsistence rates and other publicly available sources [18]. A full list of unit costs is contained in Supplemental Table 1.

The study protocol was pre-registered using the Center for Open Science registration portal (8th March 2024). Study data were collected and managed using REDCap electronic data capture tools hosted at the University of Galway [19, 20]. A descriptive statistical analysis was conducted using Microsoft Excel software to summarise the average cost estimates and the associated variation.

## Results

The survey required an average of four minutes per participant to complete. A total of 8 person-hours were required to collect the responses. Responses were successfully gathered from 42 individuals, while a further 4 declined to participate. Of the 42 participants, 37 participants completed all of the questions, while 5 provided partial responses as they were called to their appointments before the questionnaire could be completed. Overall, the questionnaire demonstrated good acceptability and feasibility. Demographic details of respondents are included in Table 1.

**Table 1:** Included participants. *N* = 42

	*N*	%
Female	26	62%
Male	14	33%
Unknown/not stated	2	5%
Age - median (IQR))	54 (46–73)	
Ethnicity - White Irish	28	67%
Ethnicity - Irish Traveller	2	5%
Ethnicity - Other White	5	10%
Ethnicity - Black or Black Irish	3	7%
Ethnicity - unknown	4	10%
Employed (FT or PT)	13	31%
Self-employed	2	5%
Student	0	0%
Unemployed or retired	21	50%
GMS card holder	27	64%
GP visit card holder	3	
Health insurance	15	36%
Education - primary	5	12%
Education - secondary	15	36%
Education - third level	19	45%
Residence - Rural	22	52%
Residence - Urban	15	36%
Married or cohabiting	21	50%
Single, Separated/divorced or widowed	18	43%

*GP: General Practitioner, GMS: General Medical Scheme (“Medical Card”) holder, FT: Full-time, PT: Part time, IQR: Inter-quartile range

To calculate the total private cost per patient, individual resource costs relating to the patient’s time, the time of their accompanying carer (if any), the cost of arranging alternative care for their dependents (if applicable) and their travel costs were combined. The median total private cost per patient of attending a hospital outpatient clinic was €131 (IQR €57-€199; range €22-€370). Participants reported attending on average 4 appointments per year (mean 4.26, range 1–20, SD 3.7), giving a mean annual total private cost of attending hospital clinics of €559 per patient. The resources used by participants are detailed in Table 2.

Participants reported spending on average 1.2 h (SD 0.72; range 0.1–3.0 h) travelling to their appointment, with a mean round-trip of 90 km (median 79 km, range 2–292 km). Fourteen participants (33%) reported missing work to attend their appointments. Many respondents reported flexible working arrangements such that they could rearrange their work around their appointment. ‘Missed work’ was defined as the respondents’ report of whether they missed work or not. Two respondents reported using their paid annual leave entitlements to attend; this was costed as equivalent to missed work as it was paid. Eighteen participants were accompanied by carers, with 4 of their carers being paid. Eight participants reported having to arrange alternative care for their dependents while they attended the appointment, though the majority of these (7) were unpaid arrangements. Of these 8 participants, 7 were women and one was of unknown sex.

**Table 2:** Resources used by respondents

Cost	*N*	Mean	Standard Deviation	Median	Inter-Quartile Range
**Patients**					
Reported total appointment time (including travel; hours)	40	3.46	1.50	3.00	3–5
Travel time (round trip; hours)	41	2.42	1.53	2.00	1–4
Cost of time	40	€ 58.29	€ 40.75	€ 49.75	€25-€71
**Accompanying Carers**					
Cost of Time	18	€ 83.07	€ 62.23	€ 74.63	€ 25-€135
Direct cost if paid	3	€ 42.33	€ 51.25	€ 25.00	€ 14-€63
**Travel**					
Travel distance (round trip, kilometres)	41	89.95	74.60	79.00	31–129
Travel modality*:					
Private car incl. parking	33	€ 38.37	€ 30.93	€ 35.12	€13-€56
Bus and/or train**	6	€ 14.00	€ 8.83	€ 14.00	€14-€17
Taxi	2	€ 11.50	€ 4.95	€ 11.50	€ 10-€13
Walking	2				
**Care for patient’s dependents**					
Time (hours)	8	5.63	7.69	2.50	2–6
Cost if paid	1	€ 100.00			

*3 participants used > 1 travel modality

**Note several participants used the Free Travel Scheme and so did not pay for public transport

The majority of participants travelled to their appointment by private car (33; 79%). Participants who utilised public transport reported a mean cost of public transport of €11.63; many participants availed of free public transport facilitated by the Free Travel Scheme.

Several participants reported an extra cost of eating out not specifically included in the questionnaire. This is not included in the totals reported here. This could be addressed in future research by applying a subsistence rate to the time spent travelling to and attending the appointment.

63% of respondents reported no financial stress due to their state of health, while the remaining 37% reported at least some financial stress. In addition, 20% of participants stated that attending appointments with health professionals was ‘quite’, ‘very’ or ‘extremely’ difficult. Of note, 10 patients (24%) reported health literacy challenges (needing assistance to read health related literature ‘Sometimes’, ‘Often’, or ‘Always’), suggesting that self-completed questionnaires may be inappropriate for some patients.

## Discussion

This study describes the development and pilot deployment of a data collection tool designed to quantify the private costs experienced by patients and their households attending outpatient clinics in hospitals in the West of Ireland. The successful pilot study demonstrated that it is possible to estimate this cost using this method, and that the questionnaire is both feasible to implement and acceptable to patients, providing a foundation for future research. The high participation rate, short average completion time and minimal assistance required underscore the acceptability of the data collection tool.

The pilot data revealed significant variability in costs between participants, reflecting the diverse characteristics of the sample. The sample for this pilot is small (42 participants), was opportunistically recruited, and is not intended to be generalisable. Furthermore, it is possible that this opportunistic sample was influenced by selection bias. A potentially important influence of gender was observed: participants who reported having to arrange alternative care for dependents while attending were overwhelmingly female, as were accompanying carers. This suggests that women may bear a disproportionate burden, which warrants further investigation in larger studies to understand and address gender-specific challenges. Existing literature suggests that women bear a disproportionate burden of unpaid caring roles internationally [21]. If this finding is borne out in future larger studies, it would have significant implications for healthcare policy and planning to ensure equitability.

PPI involvement lent particular strength to this project, reinforcing that the research question was relevant and worthwhile, and the methods appropriate for purpose. When reviewing the outcome, the PPI group thought that the results were interesting and appreciated representation of differing costs for people of different backgrounds and rural/urban dwellings, reflected that cost could be a major determinant in someone’s ability to attend appointments, asked whether results like these could be fed back to governmental and healthcare decision-makers, while also reflecting that many of the costs have complex determinants and may be difficult to mitigate (e.g. costs of private carers etc.).

In this pilot, we asked participants to estimate their total time spent at the appointment (including travel and appointment time together). This may have led to inaccurate estimates of time and future studies should ask for separate estimates for travel time and appointment time. Moreover, because the questionnaire was administered prior to the appointment, the respondents’ estimates of the appointment time may be inaccurate. Repeated measures after the appointment, or alternatively, observed studies of patients’ time and experience within the hospital environments could provide more detailed and accurate estimates of the time spent accessing care of this type. Future work could also elucidate further the costs associated with arranging alternative care for patients’ dependents.

In this study, we valued the cost of participants’ time according to their employment status and their self-report of whether they missed work to attend their appointment. This fails to account for significant other occupations and contributions by participants such as childcare or voluntary work and is a limitation of the current methodology.

Subsistence costs associated with travel (e.g. eating out) were mentioned by some respondents, but not included in this analysis. Future research should consider adding a subsistence allowance to the time spent travelling to and attending the appointment. A further limitation of this study was the exclusion of environmental, energy, and carbon costs, which are increasingly relevant to the sustainability of healthcare systems. This is particularly pertinent when comparing in-person appointments, as detailed here, to telemedicine consultations in other contexts. Future studies with larger sample sizes could address the primary drivers of patients’ financial burden and identifying which groups are most affected.

Understanding costs from the patient perspective is a critical component of comprehensive cost analyses of outpatient care. Such analyses are essential for evaluating the value and cost-effectiveness of different models of care delivery. Incorporating patients’ perspectives using patient-reported outcome measures or qualitative measures of patients perceived value of attending appointments would further enhance this work.

While there are no analogous data available with which to compare, we hope that these efforts will serve as a benchmark for further research on the private financial burden incurred by patients and their households when accessing outpatient care. Based on the pilot data, a sample size calculation for a descriptive study of a continuous variable indicates that a sample of 6,150 participants would be required to assess the costs incurred by patients with 5% precision and a 95% confidence interval. We believe that data collected in this way in a definitive study could provide important information to inform decisions around the design and optimal management of outpatient care systems.

## Conclusions

This study successfully developed and piloted a data collection tool to assess the private economic costs incurred by patients attending outpatient clinics, demonstrating its acceptability and feasibility. Though not generalisable, these findings reveal significant variability in costs, potential gender disparities, and identify key areas for future research. These insights will be valuable for researchers internationally who are interested in quantifying the financial burden faced by patients accessing healthcare. Furthermore, the findings support efforts to advance equitable and cost-effective healthcare delivery models.

## Supplementary Information

Below is the link to the electronic supplementary material.

Supplementary Material 1

Supplementary Material 2

## Data Availability

The datasets used and/or analysed during the current study are available from the corresponding author on reasonable request.
