# Supplementary material for: Assessing the financial burden on patients and their households attending hospital clinics: a pilot cross-sectional study
Source: BMC Health Serv Res. 2025 Oct 22;25:1390. doi: 10.1186/s12913-025-13503-0 (PMC12542273; doi:10.1186/s12913-025-13503-0)
Supplement: Supplementary file 1 — Supplementary Material 1 [file 12913_2025_13503_MOESM1_ESM.pdf]

Patient Cost Participant Questionnaire

A pilot study addressing the economic cost to patients attending clinic

I have read and understood the Information Leaflet about this research project. The information has been fully explained to me and I have been able to ask questions, all of which have been answered to my satisfaction.I understand that I have the right to withdraw my consent prior to starting the surveyI understand that as data is anonymised, there is no opportunity to withdraw consent following the survey.I understand that I don't have to take part in this study and that I can opt out at any time. I understand that I don't have to give a reason for opting out and I understand that opting out won't affect my future medical care.I understand that I have the right to decline to offer any particular informationI have had enough time to decide whether to participate in the survey.I consent to take part in this research study having been fully informed of the risks, benefits and alternatives.

By taking part in this survey, it will be considered that you have read and understood the information leaflet and have given consent for your responses to be processed as part of this research project.

For any queries, please contact louise.rabbitt2@hse.ie

Do you give your consent to participate in this study

- ◯ Yes
- ◯ No

Health Literacy

How often do you need to have someone help you when you read instructions, pamphlets, or other written material from your doctor or pharmacy?

- ◯ Never
- ◯ Rarely
- ◯ Sometimes
- ◯ Often
- ◯ Always

Your private expenses: Your time

How many outpatient appointments have you attended in the last 12 months? __________________________________

How many hours do you spend at the hospital for each outpatient appointment (including travelling and attending) __________________________________

How many hours did you spend traveling to today's outpatient appointment? __________________________________

What is your current employment status

- ◯ Full time employee
- ◯ Part time employee
- ◯ Unemployed
- ◯ Student
- ◯ Self-employed
- ◯ Homemaker
- ◯ Retired
- ◯ If other please specify

Other employment, please specify: __________________________________

Did you miss work to attend today's appointment?	◯ Yes<br>◯ No<br>◯ Does not apply
Do you attend school, college, or other continuing education?	◯ Yes<br>◯ No
Did you miss school or college to attend today's outpatient appointment?	◯ Yes<br>◯ No

Your private expenses: your care costs

Did you need to get someone to look after your dependents (children/child/dependent adult/pet) so you could attend today's appointment?	◯ Yes<br>◯ No<br>◯ Does not apply
How many hours do you need this carer for this appointment?	______________________
Do you pay this carer?	◯ Yes<br>◯ No
How much do you pay them per appointment?	______________________
Did you require someone else to drive or accompany you to today's appointment?	◯ Yes<br>◯ No
Do you pay this person to accompany you to appointments	◯ Yes<br>◯ No
How much do you pay them per appointment	______________________
Which of the following describes the person who accompanied you today:	◯ Works full-time<br>◯ Works part-time<br>◯ Carer<br>◯ Retired<br>◯ Other
Which of the following describes the person who accompanied you today: Other - please specify:	______________________

Travel Expenses

How many kilometers did you have to travel from your home to your outpatient appointment today?	______________________
From where did you travel today? (if kms unknown)	______________________

How did you travel to today's appointment? Please list all that apply.	☐ Bus<br>☐ Car<br>☐ Train<br>☐ Taxi<br>☐ Walking<br>☐ Other
How did you travel today?	__________________
If you drove, how much did you spend on parking today?	__________________
If you used public transport, how much did you spend to travel today?	__________________
Did you have any other expenses today that we have not mentioned?	__________________

Missed appointments

Have you ever missed an appointment because of financial or other reasons?	◯ Yes<br>◯ No
What were the reasons for missing appointments?	☐ Financial reasons<br>☐ My carer responsibilities<br>☐ Unable to get time off work<br>☐ No one available to drive or accompany me<br>☐ Other
Other reasons for missing visits - please specify:	__________________

Financial stress caused by your state of health

I feel financially stresed due to my state of health	◯ Not at all<br>◯ A little bit<br>◯ Somewhat<br>◯ Quite a bit<br>◯ Very much

Please tell us how much difficulty you have with the following:

	Not difficult	A little difficult	Quite difficult	Very difficult	Extremely difficult	Does not apply
Paying for prescriptions, over the counter medicines, and equipment	◯	◯	◯	◯	◯	◯
Attending appointments with health professionals (eg getting time off work, arranging transport, etc)	◯	◯	◯	◯	◯	◯

Demographics

Gender	◯ Female<br>◯ Male<br>◯ Other, please specify
Please specify gender	__________________________________
What is your age today (in years)	__________________________________
Do you have a medical card?	◯ Yes<br>◯ No
Do you have a GP visit card?	◯ Yes<br>◯ No
Do you have private health insurance?	◯ Yes<br>◯ No
What is the highest level of education you have completed	◯ Primary level<br>◯ Second level<br>◯ Third level<br>◯ Not applicable
Age at which you left formal education	__________________________________
What is your current marital status?	◯ Married<br>◯ Co-habiting<br>◯ Widowed<br>◯ Separated or divorced<br>◯ Single or never married
What is your total annual household income?	◯ Less than €20,000<br>◯ €20,000 to €34,999<br>◯ €35,000 to €49,999<br>◯ €50,000 to €74,999<br>◯ €75,000 to €99,999<br>◯ Over €100,000
What is your current living situation?	◯ Living alone<br>◯ Living with partner<br>◯ Living with parent(s)<br>◯ Other, please specify
Living situation: other, please specify	__________________________________
What type of accommodation do you live in?	◯ Detached<br>◯ Semi-detached/end of terrace<br>◯ Mid-terrace<br>◯ Apartment block<br>◯ Other, please specify
What kind of accommodation do you live in ?	__________________________________

Is your home	◯ Owned with mortgage<br>◯ Owned outright<br>◯ Rented privately<br>◯ Rented from council<br>◯ Other, please specify
Is your home, other please specify:	______________________
Is your home in an urban or rural area?	◯ Urban<br>◯ Rural

What is your ethnic or cultural background, please choose one:

◯ White - Irish
◯ White - Irish Traveller
◯ Any other White background
◯ Black or Black Irish - African
◯ Black or Black Irish - Any other Black background
◯ Asian or Asian Irish
◯ Asian or Asian Irish - Chinese
◯ Any other Asian background
◯ Other including mixed background,

Other ethnicity, please specify:	______________________
Notes	______________________
