# Supplementary material for: Assessing the financial burden on patients and their households attending hospital clinics: a pilot cross-sectional study
Source: BMC Health Serv Res. 2025 Oct 22;25:1390. doi: 10.1186/s12913-025-13503-0 (PMC12542273; doi:10.1186/s12913-025-13503-0)
Supplement: Supplementary file 2 — Supplementary Material 2 [file 12913_2025_13503_MOESM2_ESM.pdf]

Supplementary Table 1 – Unit costs and sources

	Unit	€	Source	Date accessed
National Average hourly wage	per hour	28.43	https://www.cso.ie/en/releasesandpublications/ep/p-elcq/earningsandlabourcostsq32023finalq42023preliminaryestimates/	8/5/2024
Employed part-time: use 50% of average wage		14.22		
Cost of leisure time and unemployed/students/retired: use 35% of national average hourly gross wage		9.95		
**Public Transport**				
Taxi fare standard rate first 0.5km	first 0.5km	4.20	https://www.transportforireland.ie/fares/taxi-fares/	8/5/2024
Taxi fare standard rate per km next 14.5km	next 14.5km	1.30	https://www.transportforireland.ie/fares/taxi-fares/	8/5/2024
City bus fare	per trip	2.20	https://www.buseireann.ie/inner.php?id=459	8/5/2024
**Parking**				
City centre on-street parking	per hour	2.00	https://www.galwaycity.ie/parking-galway-information#:~:text=Conditions%20of%20Pay%20%26%20Display%20On%2DStreet%20Parking%3A&text=Charges%20are%3A,4%20euro%20for%20two%20hours	8/5/2024
GUH parking charges	per hour	2.00	https://www.parkrite.ie/carparks/university-college-hospital-galway	8/5/2024
**Civil service travel and subsistence rates**<br>Up to 1500km (mid-point of engine size scale)	per km	0.43	https://www.revenue.ie/en/employing-people/employee-expenses/travel-and-subsistence/civil-service-rates.aspx	8/5/2024
Free Travel Scheme			https://www.citizensinformation.ie/en/social-welfare/extra-social-welfare-benefits/free-travel/	8/5/2024
